# Psychosocial factors associated with quality of life in cancer survivors: umbrella review

**DOI:** 10.1007/s00432-024-05749-8

**Published:** 2024-05-10

**Authors:** Viktorya Voskanyan, Chiara Marzorati, Diana Sala, Roberto Grasso, Ricardo Pietrobon, Iris van der Heide, Merel Engelaar, Nanne Bos, Augusto Caraceni, Norbert Couspel, Montse Ferrer, Mogens Groenvold, Stein Kaasa, Claudio Lombardo, Aude Sirven, Hugo Vachon, Galina Velikova, Cinzia Brunelli, Giovanni Apolone, Gabriella Pravettoni

**Affiliations:** 1 Applied Research Division for Cognitive and Psychological Science, IEO, European Institute of Oncology IRCCS, Milan, Italy; 2 Department of Oncology and Hemato-Oncology, University of Milan, Milan, Italy; 3 SporeData, Inc., Durham, NC USA; 4 Nivel, Netherlands Institute for Health Services Research, Utrecht, Netherlands; 5 Dipartimento Di Scienze Cliniche E Di Comunità, Università Degli Studi Di Milano, Milan, Italy; 6 European Cancer Organisation, Brussels, Belgium; 7 Health Services Research Group, Hospital del Mar Research Institute, Barcelona, Spain; 8 Department of Public Health, Bispebjerg Hospital and University of Copenhagen, Copenhagen, Denmark; 9 Department of Oncology, Oslo University Hospital, Oslo, Norway; 10 OECI-EEIG Organisation of European Cancer Institutes—European Economic Interest Grouping, Brussels, Belgium; 11 UNICANCER, Paris, France; 12 EORTC, Brussels, Belgium; 13 Leeds Institute of Medical Research at St James’s, University of Leeds, Leeds, UK; 14 Leeds Cancer Centre, Leeds Teaching Hospitals NHS Trust, Leeds, UK; 15 Scientific Directorate, Fondazione Istituto Di Ricovero E Cura a Carattere Scientifico, Fondazione IRCCS Istituto Nazionale Dei Tumori, Milan, Italy

**Keywords:** Cancer, Survivorship, Quality of life, Umbrella review

## Introduction

Cancer is a severe disease affecting millions of people worldwide (19.3 million new cases in 2020) (Sung et al. 2021). It has one of the highest mortality rates (Sung et al. 2021). However, due to advances in early cancer screening, detection, and treatment, the number of cancer survivors is rapidly increasing. Cancer survivorship has been defined in several ways, leading to different definitions (Marzorati et al. 2017). In one of the first definitions survivorship was described as a process consisting of 3 phases, acute survival phase, extended survival phase, and permanent survivorship (Mullan 1985). Later survivorship trajectories were expanded to 4 stages by adding the transitional survival phase. The “acute survival phase” starts with the diagnosis, the “transitional survival phase” is characterized by the end of treatment and the time when survivors are distancing from the medical team, the “extended survival phase” involves survivors in remission or with no evidence of disease, and the “permanent survivorship phase” begins when survivors are cancer free, but still experiencing long-term health and psychological issues (Vaz-Luis et al. 2022). However, despite the treatment and recovery, survivors still face numerous challenges, as cancer may leave a great impact on many aspects of survivors’ lives (Harrington et al. 2010). More specifically, cancer survivors face physical dysfunctions, psychological and social problems, that lead to an overall decrease in well-being and quality of life (QoL) (Ligt et al. 2019). The World Health Organization (WHO) defines QoL as “an individual's perception of their position in life in the context of the culture and value systems in which they live and in relation to their goals, expectations, standards, and concerns” (“The World Health Organization quality of life assessment (WHOQOL): Position paper from the World Health Organization” 1995). However, QoL is a complex and multifaceted concept and is defined and measured differently depending on a number of circumstances. Yet, many scientists measuring QoL follow a policy of incorporating physical function, mental status, and the ability to engage in normative social interactions (Spitzer 1987; Post 2014).

A recent study showed that overall QoL in cancer survivors has been broadly and significantly affected by psychological aspects and social support (Park et al. 2021). Indeed, psychosocial factors associated with quality of life represent a complex set of variables that impact an individual’s emotional, social, and psychological well-being. These factors depending on different circumstances (i.e., sociodemographic, clinical, cultural, etc.) can have both a positive and negative impact on survivors’ well-being and cause various modifications in QoL. The identification of psychosocial factors associated with QoL may have theoretical and clinical implications for supporting cancer survivors in their cancer journey and providing quality cancer care aimed to improve not only the clinical outcomes but also the QoL of cancer survivors. Therefore, this umbrella review(UR) highlights the importance of investigating possible moderators of QoL in cancer survivors.

Reviewing the literature, it can be stated that in recent years, given the increasing number of cancer survivors and their extended life expectancy, there is also a growing interest in QoL during this phase of cancer care. However, there is still no umbrella review compiling evidence from multiple existing reviews on psychosocial factors associated with QoL in cancer survivors. Thus, the aim of this UR is to provide a comprehensive overview of the QoL and its psychosocial determinants in cancer survivors.

## Materials and methods

### Study design

The UR was conducted following the guidelines provided by the Joanna Briggs Institute (Aromataris et al. 2015). The results are reported in accordance with Preferred Reporting Items for Systematic Reviews and Meta-Analyses (PRISMA) (Page et al. 2021). The protocol of the study is registered in the International Prospective Register of Systematic Reviews (PROSPERO, identifier: CRD42023415288) (Vokanyan et al. 2023). Therefore, the following research question was formulated: “What are the psychosocial factors associated with QoL in cancer survivors?”.

A narrative synthesis was performed to report the results.

### Data sources and search strategy

PubMed, Embase, Scopus, and PsycINFO were searched from 2012 to January 30, 2023, to identify Systematic Reviews (SRs) assessing the psychosocial factors associated with QoL in cancer survivors.

The search strategy was optimized with the assistance of a research librarian (A.V.A.). It consisted of the combination of several search terms with the following themes: Cancer, Quality of life, Factors, Psycho-social, Impact, and Survivorship. The primary search string was developed for PubMed and then modified accordingly for all other databases. The themes with their related keywords and the search strategy for each database are presented in Supplemental Table 1 and Table 2.

### Inclusion and exclusion criteria

The inclusion criteria for this UR included (1) systematic reviews (2) presenting studies on adults (over 18 years of age), (3) cancer survivors (referring to those who have completed active treatment (Marzorati et al. 2017)), (4) investigating the association between quality of life and psychosocial factors. The included papers had to be (5) written in English and (6) published between 2012 and January 2023.

The UR was designed to include both Randomized Controlled Trials (RCTs) and other types of studies (non-RCTs). This was done in order not to leave out any relevant studies on survivors where RTC design may not have been adopted.

Reviews only investigating non-psychosocial factors associated with QoL (e.g., cultural factors), as well as reviews not reporting the association between psychosocial factors and QoL were excluded.

No restriction was applied on geographical location.

### Study selection

The screening was organized in the online SR software Rayyan (Ouzzani et al. 2016). Search results were imported into Rayyan and duplicates were identified and removed. Three researchers were involved in the screening process. The preliminary screening was conducted independently by two researchers (V.V., D.S.) based on the titles, abstracts, and keywords. Researchers were blinded to each other’s decisions. Any disagreements concerning the eligibility of studies were resolved by the third researcher (C.M.) through group discussion and full-text review. All the potentially relevant articles retrieved for full-text screening were accessed using the inclusion and exclusion criteria. Any uncertainty for the final inclusion was settled through consensus. Potential conflict rates were 12.3% for the initial screening and 5.2% for the full-text screening.

### Data extraction

The following data were extracted from the retrieved articles: publication data (i.e., name of the first author, year of publication, study origin, study design), the aim of the research, characteristics of the included studies (i.e., number and type of studies included in the review, date range, and country of origin of the included studies), participants’ characteristics (i.e., sample size, socio-demographic characteristics), cancer group (i.e., cancer type and stage, time since diagnosis or treatment), factors (psychological, social), positive/negative association with QoL. Though this UR was aimed at researching psychosocial factors, the authors also collected major clinical factors that were identified in the studies researching psychosocial factors.

### Assessment of methodological quality

The methodological quality of included reviews was assessed by 2 reviewers independently using the Assessing the Methodological Quality of Systematic Reviews (AMSTAR 2) (Shea et al. 2017). Sixteen questions were applied to the included articles to evaluate the methodological quality and risk of bias of the selected studies. Each article received a score based on the number of positive, partial positive, and negative responses (the higher the rate of positive responses is, the lower the risk of bias the study has).

For this UR, AMSTAR 2 was modified: “partial yes” was added for questions, where the systematic reviews did not meet 1 criterion only for rating “Yes”; also, for question 1 regarding PICO, “Yes” was rated if the non-interventional reviews reported population and outcome only, but had a clear and predefined research question.

## Results

### Results of the selection process

**Fig. 1:**
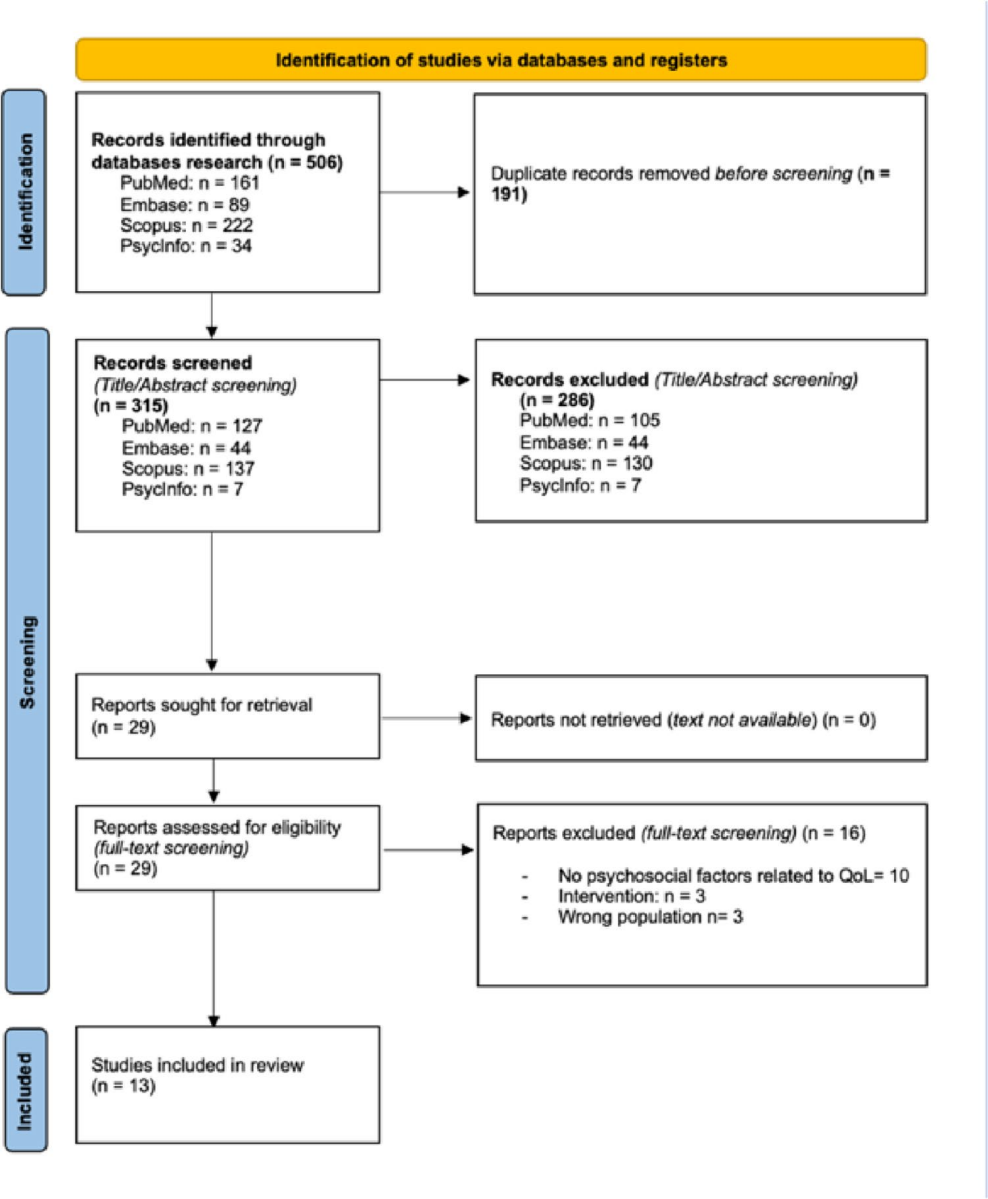
PRISMA Flowchart of the study selection process

The search in 4 electronic databases (PubMed, Embase, Scopus, and PsycINFO) identified 506 references. After the removal of the duplicates, 315 studies were selected for title and abstract screening. Twenty-nine potentially relevant reviews were retrieved for full-text screening, out of which 16 were excluded for various reasons. Thirteen articles met the inclusion criteria and were included in this UR. All the details of the selection process are summarized in Prisma Diagram Fig. 1.

### Characteristics of the included studies

This UR includes a final number of 13 systematic reviews, out of which 1 also involves a meta-analysis. The publication year range of the included studies is 2012–2022. The studies were conducted in the following countries: USA (n = 3), UK (n = 1), Malaysia (n = 1), Canada (n = 1), Spain (n = 1), Belgium (n = 1), Netherlands (n = 1), Ireland (n = 1), Denmark (n = 1), Italy (n = 1) and Germany (n = 1). In terms of cancer type, 6 studies included breast cancer survivors, 2 studies reported on colorectal cancer survivors, 4 studies focused on one cancer type each including low-grade glioma, melanoma cancer, gynecological cancer, head and neck cancer, and 1 study did not apply any restrictions on cancer type. The sample range of the included systematic reviews was 2093–36,336 participants and the mean age range was 33–82 years. Time after treatment or diagnosis time varied greatly ranging from 1 month – 20 years. The summary of the study characteristics is represented in Tables 1 and 2.

**Table 1:** Characteristics of the included studies (part 1)

Author, year of publication	Study origin	Study design	Studies included				Participants		Cancer Group		
			Number of studies included	Type of studies include	Data range of included studies	Country of origin of included studies	Sample size (N)	Socio-demographic characteristics	Cancer type	Cancer stage	Time since diagnosis or treatment
Zainal et al. (2013)	Malaysia	Systematic review	32	20 Cross-sectional studies, 10 prospective studies, 1 case control study, 1 cohort study	1998–2012	USA (n = 13), UK (n = 4), Australia (n = 2), Netherlands (n = 2), Turkey (n = 2), Norway (n = 1), Japan (n = 1), Germany (n = 1), Brazil (n = 1), China (n = 1),, Iran (n = 1), Taiwan (n = 1), Korea (n = 1), Italy (n = 1)	N = 10,826. Median sample size 129. Sample size range 15–2208	Mean age range 47—63. In the western studies the ethnic majority were Caucasians or white (median = 80%, range: 30–100%), three-quarter of the subjects were married (median = 73%, range = 33–100%)	Breast cancer	0–II	1–98 months
Howard-Anderson et al. (2012)	USA	Systematic review	28	15 Cross-sectional studies, 8 longitudinal studies, 5 RCTs	1996–2010	N/A	Sample size range 144–657	Women younger than 51 years. Mean age range 33–50	Breast cancer	N/A	< 2 month–13.2 years
Syrowatka et al. (2017)	Canada	Systematic review	42	21 Cohort studies, 20 cross-sectional studies, 1 retrospective chart review	2001–2016	USA (n = 9), Taiwan(n = 4), China(n = 3), Korea (n = 3), Sweden(n = 3), Italy(n = 2), Netherlands (n = 2), England(n = 1), Germany (n = 1), Iran (n = 1), Israel (n = 1), France (n = 1), Scotland (n = 1)	N = 20,216. Sample size range 39–2595	Mean age range 43.6–66.4. Age range 24–81	Breast cancer	0–IV	1–10 years
Rimmer et al. (2023)	England	Systematic review	29	13 Cross-sectional studies, 9 longitudinal studies	2001–2021	Netherlands (n = 3), USA (n = 3), China (n = 2), Italy (n = 2), India (n = 2), Japan (n = 2), Norway (n = 2), Australia (n = 1), Finland (n = 1), Germany (n = 1), South Korea (n = 1), Sweden (n = 1), Turkey (n = 1)	N = 2093. Sample size range 15–260	Mean age range 35.8–49.5.Sex range 24–73% female	Low-grade glioma	I–II	1–20 years
Wen et al. (2014)	USA	Systematic review	26	10 Qualitative studies, 10 quantitative studies, 5 mixed-method approach studies, 1 intervention study	1997–2013	N/A	N = 7322. Sample size range 9–206	Mean age range 33–75. Age range 29–91	Breast cancer	0–IV	0–5 years
Aizpurua-Perez et al. (2020)	Spain	Systematic review	39	30 Cross-sectional studies, 8 intervention study, 1 longitudinal study	2011–2020	Asia (n = 16), Europe (n = 14), America (n = 8), Oceania (n = 1)	Sample size range 10–540	Age range 18–90	Breast cancer	I–IV	Mean 85 days–4.9 years
Hamel et al. (2016)	Belgium	Systematic review	10	7 cross-sectional studies, 1 prospective study	2007–2015	N/A	N = 4246. Sample size range 277—1320	The mean age 55.9. 54.9% female	Melanoma cancer	N/A	Mean 1.2—9 years
Bours et al. (2016)	Netherlands	Systematic review	53	36 cross-sectional studies, 17 longitudinal studies	1994–2014	Netherlands (n = 9), USA (n = 20), Australia (7), Canada (n = 3), UK (n = 4), Greece (n = 2), Germany (n = 3), Turkey (n = 1), France (n = 1), Denmark (n = 1), Japan (n = 1), China (n = 1)	N = 36,336. Sample size range 77—1966	Mean age range 61–82	Colorectal cancer	I–IV	2–12 years postdiagnosis
Dunne et al. (2017)	Ireland	Systematic review	24	10 prospective studies, 9 cross-sectional studies	2004–2015	UK (n = 7), Norway (n = 2), USA (n = 2), Australia (n = 2), the Netherlands (n = 2), Germany (n = 1), China (n = 1), Poland (n = 1), Taiwan (n = 1)	N = 2347. Sample size range 51—376	Mean age 61. Age range 23–94. Sex 29% female	Head and neck cancer	N/A	1 month -7 years
Han et al. (2020)	USA	Systematic review and meta-analysis	35	21 cross-sectional studies, 14 longitudinal, prospective studies	2008–2019	UK (n = 9), USA (n = 9), Australia (n = 8), China (n = 3), Canada and Australia (n = 2), Taiwan (n = 1), Ireland (n = 1), Netherlands (n = 1), Portugal (n = 1),	N = 17,215	Mean age 63. Age range 50–70. The majority of participants (N = 24 out of 35 studies) were male, 2 studies included only female participants	Colorectal cancer	I–IV	< 11 years after diagnosis
Dahl et al. (2013)	Denmark	Systematic review	57	N/A	1995–2012	Scandinavia, Austria, Australia, United States, Syria, Hawaii	N/A	N/A	gynecological cancer	N/A	N/A
Koch et al. (2013)	Germany	Systematic review	17	N/A	2002–2010	USA(n = 15), Germany (n = 1), Norway(n = 1), the Netherlands(1)	N = 6352. Sample size range 92–1366	Mean age range 44.8–75.9. Age range 29–95. White/Caucasian or African American in six studies, others had no restriction on race	Breast cancer, head and neck, gynaecological, bladder, prostate, colorectal, leukaemia, non-Hodgkin's lymphoma, Wilms tumor, brain tumor, testicular	N/A	Mean 1.5—21.4 years
Durosini et al. (2022)	Italy	systematic review	33	23 cross-sectional studies, 1 longitudinal design study, 1 three-wave longitudinal study, 1 longitudinal study, 4 RCTs, 1 experimental study, 1 evidence-based interventions study, 1 ross sectional and longitudinal study	2000–2020	N/A	N = 6396	N/A	Breast cancer	N/A	N/A

**Table 2:** Characteristics of the included studies (part 2)

Author, year of Publication	Study origin	Study design	Factors		Association with QoL
			Psychological factors	Social factors	
Zainal et al. (2013)	Malaysia	Systematic review	Depression		Depression is associated with lower QoL
Howard-Anderson et al. (2012)	USA	Systematic review	Depression, stress, coping	Social support	Social and emotional support, coping are associated with better QoL
					Depressions, stress are associated with lower QoL
Syrowatka et al. (2017)	Canada	Systematic review	Distress		Distress is associated with lower QoL
Rimmer et al. (2023)	England	Systematic review	coping, depression, post-traumatic stress disorder, post-traumatic growth		Post-traumatic stress disorder, higher levels of avoidant coping, higher level of depression are associated with lower QoL. Post-traumatic growth is associated with better QoL
Wen et al. (2014)	USA	Systematic review	Emotions, emotional well-being	Social support	Negative emotions are associated with lower HRQoL. Emotional well-being and social support are associated with higher QoL
Aizpurua-Perez et al. (2020)	Spain	Systematic review	Resilience, coping strategies, anxiety, depression	Social support	Resilience, appropriate coping strategies, social support are associated with higher QoL. Anxiety/depressive symptoms are associated with lower QoL
Hamel et al. (2016)	Belgium	Systematic review		Social interactions	Social interactions are associated with higher QoL
Bours et al. (2016)	Netherlands	Systematic review	Psychological distress, anxiety, depression, optimism, cancer-threat appraisal, a sense of coherence, repression defense, benefit-finding, posttraumatic growth, faith and meaning/peace, denial, hostility	Social support	Less perceived social support, psychological distress, anxiety, depression, lower optimism and negative cancer-threat appraisal, a weaker sense of coherence, more repression defense, less benefit finding, lower posttraumatic growth, less faith and meaning/peace, and less denial and more hostility are associated with lower HRQoL
Dunne et al. (2017)	Ireland	Systematic review	Depression, anxiety, coping, fear of cancer recurrence, body image concerns, emotional and spiritual growth, neuroticism	Social support	Depression, anxiety, avoidance coping, fear of cancer recurrence, body image concerns, neuroticism are associated with lower QoL. Emotional and spiritual growth, perceptions and care received, satisfaction with social support are associated with higher QoL
Han et al. (2020)	USA	Systematic review and meta-analysis	Psychological distress, body image distress		Psychological distress, body image distress are associated with lower QoL
Dahl et al. (2013)	Denmark	Systematic review	Pessimism, fear of cancer recurrence, coping, neuroticism, sense of coherence, hope, well-being at the time of diagnosis, impaired sexual life		Hope, positive coping, high sense of coherence, positive coping, well-being at the time of diagnosis are associated with higher QoL. Pessimism, neuroticism, fear of cancer recurrence, impaired sexual life are associated with lower QoL
Koch et al. (2013)	Germany	Systematic review	Fear of cancer recurrence		Fear of cancer recurrence is significantly associated with lower QoL
Durosini et al. (2022)	Italy	Systematic review	Emotional abilities (EA), coping strategies		Active coping/EA is associated with higher QoL
					Passive coping/EA is negatively associated with lower QoL

### QoL and associated psychological factors

The results of this UR reported that the main psychological factors associated with QoL were depression, distress, and coping strategies. Depression was significantly associated with lower QoL in all the studies in which it was analyzed (Zainal et al. 2013; Howard-Anderson et al. 2012; Rimmer et al. 2023; Aizpurua-Perez and Perez-Tejada 2020; Bours et al. 2016; Dunne et al. 2017). Moreover, findings highlight that younger breast cancer survivors (< 50 years) compared to older breast cancer survivors (> 50 years) or age-matched women without cancer were more likely to experience depression or depressive symptoms (Howard-Anderson et al. 2012), which was reported to be common in survivorship, and contributed to reduced QoL (Aizpurua-Perez and Perez-Tejada 2020). Another prominent factor significantly associated with QoL was distress (Howard-Anderson et al. 2012; Bours et al. 2016; Hamel et al. 2016; Han et al. 2020; Syrowatka et al. 2017). Distress was analyzed in different domains, especially related to body image and psychological well-being, and the findings of the studies provide evidence of the negative impact of distress on QoL. Furthermore, Bours et al. outlined the long-term impact of psychological distress reporting that colorectal cancer survivors with higher levels of psychological distress demonstrated poor QoL up to 5 years of post-treatment period (Bours et al. 2016). Reviews also highlight the correlation between QoL and coping. Studies show that active coping is associated with higher QoL (Howard-Anderson et al. 2012; Rimmer et al. 2023; Dahl et al. 2013**),** whereas passive and avoidance coping in their turn have a negative impact on QoL (Dunne et al. 2017; Durosini et al. 2022). Additionally, appropriate coping strategies reduce distress and have a positive impact on QoL (Aizpurua-Perez and Perez-Tejada 2020).

The findings also underline the importance of other psychological factors associated with QoL, such as emotions, anxiety, and fear of cancer recurrence. Emotional growth and emotional abilities were associated with higher mental health-related QoL (Dunne et al. 2017; Durosini et al. 2022). These results are also confirmed by Wen et al. who reported that negative emotions were identified as barriers to good QoL by Chinese and Korean American breast cancer survivors (Wen et al. 2014). The relationship between anxiety and QoL was analyzed in three studies. The reviews demonstrated the correlation between higher levels of anxiety and lower QoL (Aizpurua-Perez and Perez-Tejada 2020; Bours et al. 2016; Dunne et al. 2017). Interestingly, fear of cancer recurrence was another factor impacting long-term QoL (Dunne et al. 2017; Dahl et al. 2013; Koch et al. 2013). Koch et al. investigating fear of cancer recurrence in long-term cancer survivors (≥ 5 years) reported its significant association with lower QoL even during prolonged survival time (Koch et al. 2013).

Finally, the findings of this UR provide evidence of other psychological factors impacting QoL. Results demonstrate that poor QoL was also associated with lower post-traumatic growth, a weaker sense of coherence, and neuroticism (Rimmer et al. 2023; Bours et al. 2016; Dunne et al. 2017). Conversely, among the factors having a positive impact on QoL were resilience, optimism, and faith/spiritual growth (Aizpurua-Perez and Perez-Tejada 2020; Bours et al. 2016; Dunne et al. 2017).

### QoL and associated social factors

On the contrary with psychological factors, there were not many social factors identified in the association with QoL. Social support and social functioning were the main factors impacting QoL (Howard-Anderson et al. 2012; Aizpurua-Perez and Perez-Tejada 2020; Bours et al. 2016; Dunne et al. 2017; Hamel et al. 2016; Wen et al. 2014). Results demonstrate that social support is an important factor in improving the negative effects of QoL (Wen et al. 2014). Specifically, greater social support and satisfaction with social support have a positive influence on QoL (Howard-Anderson et al. 2012; Wen et al. 2014), whereas lack of social support contributes to reduced QoL (Bours et al. 2016). Interestingly, the negative impact of less perceived social support is reported even in long-term survivorship. Results outline that colorectal cancer survivors with less perceived social support and worse social network measures had lower HRQoL even after 5 years of post-diagnosis (Bours et al. 2016).

### Clinical factors and their association with QoL

While investigating psychosocial factors associated with QoL in cancer survivors, this UR also extracted some major clinical factors reported in the systematic reviews. Indeed, most of the included systematic reviews refer to cancer survivorship as “a process starting from the time of diagnosis” and highlight the importance of medical aspects on QoL as well. Specifically, our findings demonstrate that comorbidities, adverse symptomology, cancer and treatment-related symptoms have been negatively associated with QoL (Howard-Anderson et al. 2012; Rimmer et al. 2023; Bours et al. 2016; Hamel et al. 2016; Han et al. 2020). Interestingly, contradictory results were highlighted regarding the association between QoL and tumor grade, tumor location, adjuvant therapy, and time after treatment. Some findings identified that tumor grade, tumor location, and adjuvant therapy have a negative impact on QoL, in contrast to other results reporting no significant association between higher tumor stage/localization, adjuvant therapy, and QoL (Bours et al. 2016). This outcome is directly in line with the study outlining that long-term QoL is not impaired with low-stage gynecologic cancer (Dahl et al. 2013). Furthermore, results concerning the impact of time after diagnosis or treatment on QoL should also be interpreted with caution, as diverse associations were found between QoL and treatment/diagnosis time-related variables. Some findings provide evidence that recent diagnosis, 1- and 3-years since treatment, and extension of time since diagnosis have a negative impact on QoL (Rimmer et al. 2023; Hamel et al. 2016). Contrary to these results, one study highlighted contrasting results regarding the influence of time after diagnosis or treatment on QoL. It reported finding both positive and negative, as well as neutral associations between QoL and time after treatment (Dahl et al. 2013).

In addition to the findings mentioned above, there were also other clinical factors negatively impacting QoL: epilepsy/seizure burden, worse Tumor-Nodes-Metastasis (TNM), more extensive surgery, tumor recurrence, fatigue, and short-term surgical complications (Rimmer et al. 2023; Bours et al. 2016; Hamel et al. 2016; Dahl et al. 2013).

### Quality assessment

The results of the quality assessment are represented in the Supplemental Fig. 1. The main questions where most of the included systematic reviews failed regarded the registration of the protocol, the explanation of the selection of the study design, the provision of the list of the excluded studies, and the report on the sources of income of the included studies. Of all the studies included in this UR, only Rimmer et al.’s systematic review had a registered protocol (Rimmer et al. 2023), and Howard-Anderson et al.’s study reported the source of funding of only some studies included in the review (Howard-Anderson et al. 2012).

## Discussion

To our knowledge, this is the first UR identifying psychosocial factors associated with QoL in cancer survivors. The current review is based on the findings of 13 systematic reviews examining the associations between psychosocial factors and QoL in cancer survivors. More specifically, the positive and negative impacts of the psychosocial factors on QoL were carefully examined.

Overall, the results of our review confirm that QoL in cancer survivors is correlated with a considerable number of psychosocial factors.

Summarizing the findings of this UR it can be stated that the most common factors negatively and positively impacting QoL in cancer survivors are depression and social support, respectively: social support is reported to improve QoL, whereas depression is always correlated with poor QoL. This outcome ties well with the analyzed studies published within the last 10 years reporting social support and depression having a profound effect on QoL (Zainal et al. 2013; Howard-Anderson et al. 2012; Rimmer et al. 2023; Aizpurua-Perez and Perez-Tejada 2020; Bours et al. 2016; Dunne et al. 2017; Hamel et al. 2016; Wen et al. 2014). The importance of social support can be explained by the numerous negative effects caused by the disease and by the inability of cancer survivors to handle challenges alone. Social support, described as a process involving interactions between a recipient of help and individuals or entities providing it (Roberts et al. 1999), has been demonstrated as a key factor for cancer patients and survivors because it affects the adaptation to their new condition(Osann et al. 2014); family members, relatives, or friends would be present not only in daily life circumstances but also during crises throughout individuals’ lives, thus enhancing QoL for this cancer population (Ruiz-Rodríguez et al. 2022).

On the other hand, the lack of social support relates to higher numbers of anxiety and depression cases (Hu et al. 2018), which leads to lower QoL (Bours et al. 2016). Research confirms that even after the treatment survivors still face various psychological and physical/clinical issues, such as psychological distress, anxiety, depression, musculoskeletal problems, or lack of stamina (Stein et al. 2008; Agostinelli et al. 2022), which lead to reduced QoL. Therefore, survivors emphasize the important role of social support, especially from their partners, in overcoming different challenges they face, and improving overall well-being (Ruiz-Rodríguez et al. 2022; Pfaendler et al. 2015). Providing survivors with a strong “support system” or teaching them how to build one is crucial for improving their overall psychological well-being and QoL. For the cited reasons social support is strictly positively related to QoL and needs to be improved and strengthened within cancer patients (Harms et al. 2019).

Some studies have demonstrated that cancer diagnosis could activate psychological and emotional responses that can persist for years after treatment (Meyerowitz et al. 2008). The most common negative psychological long-term or late effects attributable to the cancer experience are distress, depression, and anxiety (Agostinelli et al. 2022). Thus, while some survivors can easily overcome these challenges, others struggle with emotional adjustment during the treatment and survivorship period. For this reason, addressing these mood concerns is crucial because they can disrupt survivors’ QoL and prevent them from returning to usual activities (Yi and Syrjala 2017).

In line with that, our findings demonstrate the important role of depression, distress, and stress in QoL outcomes of cancer survivors. The mentioned factors were reported as being a major cause of a decrease in QoL in a number of studies, highlighting a prevalence of these symptoms in cancer survivors, even five or more years after diagnosis (Brandenbarg et al. 2019). Specifically, six studies included in the present UR mentioned the negative impact of depression on QoL (Zainal et al. 2013; Howard-Anderson et al. 2012; Rimmer et al. 2023; Aizpurua-Perez and Perez-Tejada 2020; Bours et al. 2016; Dunne et al. 2017), and another four reviews revealed distress and stress among crucial factors responsible for poor QoL (Howard-Anderson et al. 2012; Bours et al. 2016; Han et al. 2020; Syrowatka et al. 2017). Distress and depression may have a significant impact on QoL for several reasons. Firstly, the emotional and psychological responses accompanying cancer diagnosis and treatment can lead to depressive moods and higher distress levels. Furthermore, the fear of recurrence, the scheduled follow-up surveillance necessary after a cancer diagnosis, and the perceived sense of isolation generally perceived by a cancer patient may negatively impact survivors’ QoL and psychological status, increasing levels of distress in this cancer group. Stated that, it is also possible that psychological distress arises in this population due to patients’ condition of survivorship itself [56]; indeed, the National Comprehensive Cancer Network distress guidelines describe distress as a dimension spanning on a continuum, encompassing common feelings such as vulnerability, sadness, and fear of recurrence to more severe manifestations like depression, anxiety, trauma, panic, and existential crisis (Yi and Syrjala 2017).

The findings also confirm that cancer significantly impacts survivors' emotional state and mental health, which subsequently leads to significant alterations in QoL (Dunne et al. 2017; Hamel et al. 2016; Durosini et al. 2022; Wen et al. 2014). Research shows that emotional challenges of cancer diagnosis and treatment such as anxiety, worry about the future, and a fear of cancer recurrence, are common even throughout various phases of survivorship (Aizpurua-Perez and Perez-Tejada 2020; Dunne et al. 2017). More specifically, studies demonstrate that fear of cancer recurrence is reported to have a profound impact on the QoL of cancer survivors (Dunne et al. 2017; Koch et al. 2013; Zhang et al. 2022; Rha et al. 2022; Vandraas et al. 2021; Tran et al. 2022). A recent study by Rha et al. reported that about 66% of breast cancer survivors were experiencing clinical levels of fear of cancer recurrence (Rha et al. 2022), showing how the fear of cancer recurrence is still experienced by cancer survivors long after the treatment and recovery. As reported by Tran et al. breast cancer survivors still experience a high level of fear of cancer recurrence even after 10 years from diagnosis (Tran et al. 2022). This may mean that the traumatic challenges caused by cancer diagnosis and treatment leave a long-term negative impact on cancer survivors' psychological state that causes anxiety about a potential return of cancer. This persistence of fear of cancer recurrence is noteworthy and an important issue to pay attention to, as it has its negative consequences on QoL. Moreover, fear of cancer recurrence appears to be associated not only with poor QoL, but also with depression, emotional distress, anxiety, fatigue, and trouble sleeping (Vandraas et al. 2021; Nahm et al. 2021). A possible explanation for it may be that the fear of cancer recurrence creates worry about the future and uncertainty, which leads to different mental issues and leaves a negative impact on survivors’ overall well-being (Vandraas et al. 2021; Thewes et al. 2012; Durazo and Cameron 2019). The constant worry about the future may increase the risks of anxiety, depression, and fatigue, causing restlessness and difficulty in sleeping.

Moreover, survivors generally face many post-treatment experiences with physical and psychosocial consequences and challenges, obliging survivors to numerous adaptations to different physical and mental conditions; all these could result in heightened vulnerability that, in turn, could improve levels of depression. Indeed, Zainal et al.’s study on the prevalence of depression reported breast cancer survivors being at high risk for depression and found a significant correlation between depression and QoL. Interestingly, depression has been associated not only with lower QoL, but also with some other socio-demographic variables, cancer, and treatment-related factors (Zainal et al. 2013). These findings are in line with Kim et al.’s results highlighting the correlation between depression and sociodemographic factors, comorbidity, and symptom characteristics (Kim et al. 2008). Indeed, a significant association between depression and pain, insomnia, social support, and optimism, as well as some demographic factors such as age, income, and education are reported in many studies (Kim et al. 2008; Galiano-Castillo et al. 2014).

The results of the present UR also emphasize the important role of coping strategies in influencing the QoL of cancer survivors. Coping mechanisms can serve as a powerful tool for dealing with/managing emotional and psychological challenges that cancer survivors are facing. Generally, active coping is considered the use of energy to change the circumstances causing stress, to seek social support or professional help, and to manage problems (Gao et al. 2021). Obviously, such activities can contribute to higher QoL. Indeed, findings confirm the positive influence of adopting appropriate coping strategies on increased QoL (Aizpurua-Perez and Perez-Tejada 2020), in contrast to the negative effect of avoidance coping on QoL (Rimmer et al. 2023; Dunne et al. 2017). Furthermore, the chosen type of coping strategy will contribute not only to patients’ well-being, but will also play a critical role in the degree of post-traumatic growth: acceptance and planning coping strategies have been correlated with significantly increased post-traumatic growth, and avoidant coping decreased post-traumatic growth (Nik Jaafar et al. 2021). It can thus be reasonably assumed that coping strategies aimed at acceptance and planning can provide survivors skills to face the challenges of cancer, and effectively cope with a number of psychological issues they experience.

## Limitations and future directions

The UR identified some limitations. Firstly, only studies published in English were included in our review, which leads to the possibility of having left out additional relevant reviews on this topic and country-specific characteristics. Additionally, no restrictions were applied to the cancer stage, and post-treatment/follow-up period. Survivors of advanced cancer stages or right after the completion of the treatment may experience psychosocial factors impacting their QoL that are specific just for their condition, as they are generally characterized by *worse* deteriorations in QoL in comparison with other survivor population groups. Thus, these variations in QoL that depend on different factors may explain the heterogeneity of the results.

Finally, studies included in this UR were heterogeneous in terms of cancer and treatment types. The impact of specific cancer and treatment on QoL also differs depending on the cancer type and treatment option, for example, breast cancer survivors who have undergone mastectomy may experience cancer and treatment-specific QoL issues that can not be applied to the survivors of other cancer types.

However, despite the possible limitations, this UR provides a good contribution to the QoL concept in cancer survivors and gives a broader overview of the QoL throughout survivorship trajectories.

To sum up, we can state that the summarized results demonstrate strong evidence of psychosocial factors impacting the QoL of cancer survivors, giving a clear picture of the QoL challenges they face. However, QoL is a complex phenomenon, the interpretation of which may vary greatly due to the factors mentioned in the above paragraphs. Thus, this UR encourages further research on QoL in cancer survivors from different angles and perspectives such as the impact of (1) the cancer stage, (2) the post-treatment time, and (3) treatment type.

## Clinical implications

Nowadays, there is massive evidence of the efficiency of personalized interventions, and cancer-specific self-management platforms for cancer patients and survivors (Kondylakis, et al. 2013, 2017). A broader conception of QoL and its determinants will contribute to the development of more patient-centered care aiming to reduce both physical and psychological outcomes of cancer survivors. Our findings can be a prominent base for designing and developing QoL questionnaires and instruments, tailored interventions, and policies aimed at supporting cancer survivors through all the stages of survivorship pathway.

## Conclusion

The results of this UR demonstrate that the QoL in cancer survivors is correlated with a variety of psychosocial factors. The UR identified the negative and positive influence of these determinants on QoL in cancer survivors. However, the UR highlights a need for further research on QoL in order to investigate further the concept of Qol in cancer survivors and to identify the dependencies of its associations.

Understanding the psychosocial factors associated with QoL is an important step for improving the QoL in cancer survivors, which is essential for stabilizing their overall well-being and life satisfaction.

The review was conducted on behalf of the EUonQoL Consortium.

## Supplementary Information

Below is the link to the electronic supplementary material.

Supplementary file1 (DOCX 15 KB)

Supplementary file2 (DOCX 15 KB)

## Data Availability

This request is not applicable for Umbrella Review. The tables with all the available information are already presented in the text.
