# Supplementary material for: Psychosocial factors associated with quality of life in cancer survivors: umbrella review
Source: J Cancer Res Clin Oncol. 2024 May 10;150(5):249. doi: 10.1007/s00432-024-05749-8 (PMC11087342; doi:10.1007/s00432-024-05749-8)
Supplement: Supplementary file 1 — Supplementary file1 (DOCX 15 KB) [file 432_2024_5749_MOESM1_ESM.docx]

**Supplemental Figure 1**

**Assessment of the Methodological Quality**

**AMSTAR-2 :16 items**

| **Author(s)** | **Q 1** | **Q 2** | **Q 3** | **Q 4** | **Q 5** | **Q 6** | **Q 7** | **Q 8** | **Q 9** | **Q 10** | **Q 11** | **Q 12** | **Q 13** | **Q 14** | **Q 15** | **Q 16** |
| --- | --- | --- | --- | --- | --- | --- | --- | --- | --- | --- | --- | --- | --- | --- | --- | --- |
| Zainal et al. |  |  |  |  |  |  |  |  |  |  |  |  |  |  |  |  |
| Howard-Anderson et al. |  |  |  |  |  |  |  |  |  |  |  |  |  |  |  |  |
| Syrowatka et al. |  |  |  |  |  |  |  |  |  |  |  |  |  |  |  |  |
| Rimmer et al. |  |  |  |  |  |  |  |  |  |  |  |  |  |  |  |  |
| Wen et al. |  |  |  |  |  |  |  |  |  |  |  |  |  |  |  |  |
| Aizpurua-Perez et al. |  |  |  |  |  |  |  |  |  |  |  |  |  |  |  |  |
| Hamel et al. |  |  |  |  |  |  |  |  |  |  |  |  |  |  |  |  |
| Bours et al. |  |  |  |  |  |  |  |  |  |  |  |  |  |  |  |  |
| Dunne et al. |  |  |  |  |  |  |  |  |  |  |  |  |  |  |  |  |
| J. Han et al. |  |  |  |  |  |  |  |  |  |  |  |  |  |  |  |  |
| Dahl et al. |  |  |  |  |  |  |  |  |  |  |  |  |  |  |  |  |
| Koch et al. |  |  |  |  |  |  |  |  |  |  |  |  |  |  |  |  |
| Durosini et al. |  |  |  |  |  |  |  |  |  |  |  |  |  |  |  |  |

Note: Green = yes, orange = partial yes, red = no, grey = no meta-analysis conducted.
