# Supplementary material for: Psychosocial factors associated with quality of life in cancer survivors: umbrella review
Source: J Cancer Res Clin Oncol. 2024 May 10;150(5):249. doi: 10.1007/s00432-024-05749-8 (PMC11087342; doi:10.1007/s00432-024-05749-8)
Supplement: Supplementary file 2 — Supplementary file2 (DOCX 15 KB) [file 432_2024_5749_MOESM2_ESM.docx]

**Supplemental Materials**

**Supplemental Table 1**

**Search terms and themes used in the search strategy**

| Cancer | Neoplasms  Tumor/tumour*  malignanc*  neoplasm*  carcinoma*  Oncolog* |
| --- | --- |
| Quality of life | Health-Related Quality Of Life  HRQOL  QOL |
| Factors | Factor*  idicator*  Characteristic*  predictor*  determinant* |
| Psychosocial | Psychology  Psycho*  Individual  Personal  Psychosocial Factors |
| Impact | Affect  Effect  Related  Impact |
| Survivorship/survivor | Survivor* survivorship survival* |

**Supplemental Table 2**

**Search strings for each database**

| Database | Search string |
| --- | --- |
| PubMed | ("Neoplasms"[MeSH Terms] OR cancer*[tw] OR tumor*[tw] OR tumour* [tw] OR malignanc*[tw] OR neoplasm*[tw] OR carcinoma*[tw] OR Oncolog*[tw]) AND ("Quality of Life/psychology"[Mesh] OR "Quality of Life"[Mesh] OR “Quality of life”[tw] OR “Health-Related Quality Of Life”[tw] OR “Health Related Quality Of Life”[tw] OR HRQOL[tw] OR QOL[tw]) AND (Factor*[tw] OR indicator*[tw] OR Characteristic*[tw] OR predictor*[tw] OR determinant*[tw]) AND ("psychology" [Subheading] OR "Psychology"[Mesh] OR "Neoplasms/psychology"[Mesh] OR psycho*[tw] OR social[tw] OR individual[tw] OR personal[tw] OR “Psychological Side Effect”[tw] OR “Psychological Side Effects”[tw] OR “Psychosocial Factors”[tw] OR “Factor, Psychosocial”[tw] OR “Factors, Psychosocial”[tw] OR “Psychosocial Factor”[tw] OR “Psychological Factors”[tw] OR “Factor, Psychological”[tw] OR “Psychological Factor”[tw] “Factors, Psychological”[tw] OR affect[tw] OR effect[tw] OR related[tw] OR impact[tw] OR “side effect*”[tw]) AND ("Cancer Survivors"[Mesh] OR "Survivors"[Mesh] OR “Cancer Survivor”[tw] OR “Survivors, Cancer”[tw] OR “Survivors of Childhood Cancer”[tw] OR “Childhood Cancer Survivor*”[tw] OR “Cancer Survivorship”[tw] OR “Survivorship, Cancer”[tw] OR “Long-Term Cancer Survivors”[tw] OR “Cancer Survivor, Long-Term”[tw] OR “Long Term Cancer Survivor*”[tw] OR "Survivors"[Mesh] OR Survivor[tw] OR “Long-Term Survivors”[tw] OR “Long Term Survivors”[tw] OR “Long-Term Survivor”[tw] OR “Survivor, Long-Term”[tw] OR “Survivors, Long-Term”[tw] OR survivorship[tw]) AND (systematic review[pt] OR “systematic review”[tw]) AND (2013/01/01:2022/12/31[Date - Publication] AND "english"[Language] NOT ("animals"[MeSH Terms] NOT "humans"[MeSH Terms])) |
| Scopus | INDEXTERMS(Neoplasms) OR TITLE-ABS-KEY(cancer*) OR TITLE-ABS-KEY(tumor*) OR TITLE-ABS-KEY(tumour*) OR TITLE-ABS-KEY(malignanc*) OR TITLE-ABS-KEY(neoplasm*) OR TITLE-ABS-KEY(carcinoma*) OR TITLE-ABS-KEY(Oncolog*)) AND (INDEXTERMS("Quality of Life") OR TITLE-ABS-KEY("Quality of life") OR TITLE-ABS-KEY("Health Related Quality Of Life") OR TITLE-ABS-KEY(HRQOL) OR TITLE-ABS-KEY(QOL)) AND (TITLE-ABS-KEY(Factor*) OR TITLE-ABS-KEY(indicator*) OR TITLE-ABS-KEY(Characteristic*) OR TITLE-ABS-KEY(predictor*) OR TITLE-ABS-KEY(determinant*)) AND (INDEXTERMS(Psychology) OR TITLE-ABS-KEY(psycho*) OR TITLE-ABS-KEY(social) OR TITLE-ABS-KEY(individual) OR TITLE-ABS-KEY(personal) OR TITLE-ABS-KEY("Psychological Side Effect") OR TITLE-ABS-KEY("Psychosocial Factor") OR TITLE-ABS-KEY("Factor, Psychosocial") OR TITLE-ABS-KEY("Psychological Factors") OR TITLE-ABS-KEY("Factor, Psychological") OR TITLE-ABS-KEY(affect) OR TITLE-ABS-KEY(effect) OR TITLE-ABS-KEY(related) OR TITLE-ABS-KEY(impact) OR TITLE-ABS-KEY("side effect")) AND (INDEXTERMS("Cancer Survivor") OR INDEXTERMS(Survivors) OR TITLE-ABS-KEY("Cancer Survivor") OR TITLE-ABS-KEY("Survivors, Cancer") OR TITLE-ABS-KEY("Cancer Survivorship") OR TITLE-ABS-KEY("Survivorship, Cancer") OR TITLE-ABS-KEY("Long Term Cancer Survivor") OR TITLE-ABS-KEY("Cancer Survivor, Long-Term") OR TITLE-ABS-KEY(Survivor) OR TITLE-ABS-KEY("Long Term Survivor") OR TITLE-ABS-KEY("Survivor, Long-Term") OR TITLE-ABS-KEY(survivorship)) AND ((KEY({systematic review}) OR TITLE({systematic review})) AND DOCTYPE(“Re”))  AND  LANGUAGE(english) |
| Embase | ('neoplasm'/exp OR cancer*:ti,ab,kw OR tumor*:ti,ab,kw OR tumour*:ti,ab,kw OR malignanc*:ti,ab,kw OR neoplasm*:ti,ab,kw OR carcinoma*:ti,ab,kw OR Oncolog*:ti,ab,kw) AND ('quality of life'/exp OR 'Quality of life':ti,ab,kw OR 'Health-Related Quality Of Life':ti,ab,kw OR 'Health Related Quality Of Life':ti,ab,kw OR HRQOL:ti,ab,kw OR QOL:ti,ab,kw OR 'Life quality':ti,ab,kw) AND (factor*:ti,ab,kw OR indicator*:ti,ab,kw OR characteristic*:ti,ab,kw OR predictor*:ti,ab,kw OR determinant*:ti,ab,kw) AND ('psychology'/exp OR psycho*:ti,ab,kw OR social:ti,ab,kw OR individual:ti,ab,kw OR personal:ti,ab,kw OR 'Psychological Side Effect':ti,ab,kw OR 'Psychological Side Effects':ti,ab,kw OR 'Psychosocial Factors':ti,ab,kw OR 'Factor, Psychosocial':ti,ab,kw OR 'Factors, Psychosocial':ti,ab,kw OR 'Psychosocial Factor':ti,ab,kw OR 'Psychological Factors':ti,ab,kw OR 'Factor, Psychological':ti,ab,kw OR 'Psychological Factor':ti,ab,kw 'Factors, Psychological':ti,ab,kw OR affect:ti,ab,kw OR effect:ti,ab,kw OR related:ti,ab,kw OR impact:ti,ab,kw OR 'side effect*':ti,ab,kw) AND ('cancer survivor'/exp OR 'survivor'/exp OR “Cancer Survivor”:ti,ab,kw OR “Survivors, Cancer”:ti,ab,kw OR “Cancer Survivorship”:ti,ab,kw OR “Survivorship, Cancer”:ti,ab,kw OR “Long-Term Cancer Survivors”:ti,ab,kw OR “Cancer Survivor, Long-Term”:ti,ab,kw OR “Long Term Cancer Survivor*”:ti,ab,kw OR Survivor:ti,ab,kw OR “Long-Term Survivors”:ti,ab,kw OR “Long Term Survivors”:ti,ab,kw OR “Long-Term Survivor”:ti,ab,kw OR “Survivor, Long-Term”:ti,ab,kw OR “Survivors, Long-Term”:ti,ab,kw OR survivorship:ti,ab,kw) AND (“systematic review”:pt OR 'systematic review'/mj OR 'systematic review':ti,ab,kw) NOT ('animal'/exp NOT 'human'/exp) AND [2013-2022]/py |
| PsycInfo | (exp Neoplasms/ OR cancer*.ti,ab,id. OR tumor*.ti,ab,id. OR tumour*.ti,ab,id. OR malignanc*.ti,ab,id. OR neoplasm*.ti,ab,id. OR carcinoma*.ti,ab,id. OR Oncolog*.ti,ab,id.)  AND (exp "Quality of Life"/ OR "Quality of life".ti,ab,id. OR "Health-Related Quality Of Life".ti,ab,id. OR "Health Related Quality Of Life".ti,ab,id. OR HRQOL.ti,ab,id. OR QOL.ti,ab,id.) AND (Factor*.ti,ab,id. OR indicator*.ti,ab,id. OR Characteristic*.ti,ab,id. OR predictor*.ti,ab,id. OR determinant*.ti,ab,id.) AND  (exp Psychology/ OR psycho*.ti,ab,id. OR social.ti,ab,id. OR individual.ti,ab,id. OR personal.ti,ab,id. OR "Psychological Side Effect".ti,ab,id. OR "Psychological Side Effects".ti,ab,id. OR "Psychosocial Factors".ti,ab,id. OR "Factor, Psychosocial".ti,ab,id. OR "Factors, Psychosocial".ti,ab,id. OR "Psychosocial Factor".ti,ab,id. OR "Psychological Factors".ti,ab,id. OR "Factor, Psychological".ti,ab,id. OR "Psychological Factor “Factors, Psychological”".ti,ab,id. OR affect.ti,ab,id. OR effect.ti,ab,id. OR related.ti,ab,id. OR impact.ti,ab,id. OR "side effect*".ti,ab,id.) AND  (exp Survivors/ OR "Cancer Survivor".ti,ab,id. OR "Survivors, Cancer".ti,ab,id. OR "Cancer Survivorship".ti,ab,id. OR "Survivorship, Cancer".ti,ab,id. OR "Long-Term Cancer Survivors".ti,ab,id. OR "Cancer Survivor, Long-Term".ti,ab,id. OR "Long Term Cancer Survivor*".ti,ab,id. OR Survivor.ti,ab,id. OR "Long-Term Survivors".ti,ab,id. OR "Long Term Survivors".ti,ab,id. OR "Long-Term Survivor".ti,ab,id. OR "Survivor, Long-Term".ti,ab,id. OR "Survivors, Long-Term".ti,ab,id. OR survivorship.ti,ab,id.)  AND ("systematic review".dt. OR "systematic review".pt. OR "systematic review".ti,ab,id.) |
